# Supplementary material for: Children’s Health, Wellbeing and Academic Outcomes over the Summer Holidays: A Scoping Review
Source: Children (Basel). 2024 Feb 27;11(3):287. doi: 10.3390/children11030287 (PMC10969660; doi:10.3390/children11030287)
Supplement: Supplementary file 1 [file children-11-00287-s001.zip › children-2887922-supplementary.pdf]

Supplemental Files

Table S1. study inclusion and exclusion criteria.

	**Inclusion Criteria:** *The study must meet all of the inclusion criteria below*	**Exclusion Criteria:** If the study meets any 1 of the exclusion criteria, it will be excluded
Population	Children aged 5 – 15 (25% or more of the participants fall within this age group).	Greater than 25% of the study participants outside the 5 – 18 year age range (inclusive). Studies will NOT be excluded on the basis of population characteristics related to disadvantage.
Concept	Health will be considered through a holistic lens: health outcomes and behaviours that measure any aspect of physical, mental, cognitive, emotional, social health or wellbeing will be considered. Academic outcomes will also be included.	
Context	The study must aim to understand the impact of the summer holidays.	Investigating the impact of other time periods, not including the summer holidays. E.g. impact of COVID-19, impact of other summer holidays not specific to summer holidays.
Study Design	Quantitative studies: observational (e.g., cohort, cross sectional) with repeated measures *or* a comparison group. Interventional studies (control group data only). Two measures must be included of the outcome to indicate pre/post summer changes or how the outcome differed from the school year.	Qualitative studies. Not a peer-reviewed journal article. Single, cross-sectional measures of the outcome.
Location /Language	English version or English translation available. No geographical limit.	No English version available.
Outcomes	Any health or health behaviour outcome, using any measure or instrument. **Main outcome:** Health and wellbeing outcomes, health behaviour, academic outcomes. **Additional outcomes:** Findings reported according to disadvantage (SES, race/ethnicity, other).	

Table S2. Medline search strategy and terms.

**Term**	**MeSH (Medline)**	**Keywords**
Children	child/	(child* or adolescen* or student or grade or boy or girl).ti,ab,kf.
Adolescents	adolescent/	
Summer Holidays	Holidays/	(summer* adj2 (holiday* or vacation* or school*)).ti,ab,kf.

NOT	9 (COVID-19 or pandemic or Sars-CoV-2).ti,ab,kf.	

Table S3. Comparative Outcomes for Disadvantaged Children over Summer.

**Outcome category, (n=), measure of disadvantage**	**Better for Disadvantaged Children**	**Mixed Results/No Difference for Disadvantaged Children**	**Worse for Disadvantaged Children**
**Academic Outcomes** (n=30)<br>n=25 SES<br>n= 5 race	**Academic outcomes** [53,107]	**Academic outcomes** [17,30,34,44,48,57,63,65,66,84]	**Academic outcomes** [24,28,29,42,70-72,78,79,81,82,91,97,98,102,103,108,109]
**Physical Health** (n=10)<br>n=6 SES<br>n= 4 race		**Adiposity** [9,39,52,74,96]<br>**Cardiovascular fitness** [52]	**Adiposity** [64,73,98]<br>**Cardiovascular fitness** [49]
**Health Behaviours** (n=6)<br>n=4 SES<br>n=2 race			**Screen time** [50,55,88]<br>**Physical activity** [49,55]<br>**Sleep** [55]
**Social, Emotional, Mental Wellbeing** (n=3)<br>n=3 SES		**Social-emotional wellbeing** [41,87,88]	

Legend: *n*=: number of outcomes available as stratified by disadvantage. *Measure of disadvantage*: number of studies for using SES to indicate disadvantage (measured at the individual, school or area level) or measures of race/ethnicity (when SES was not available).
